# A 5 year result of neglected posterior hip dislocation with unusual presentation of false acetabulum treat with skeletal traction followed by cemented total hip replacement: A case report

**DOI:** 10.1016/j.ijscr.2024.110813

**Published:** 2024-12-31

**Authors:** Andriessanto Ceelvin Lengkong, Albertus Djarot Noersasongko, Haryanto Karmansyah Sunaryo, Rangga B.V. Rawung, Stefan A.G.P. Kambey, Alfons Datui

**Affiliations:** a Division of Orthopaedic Surgery, Department of Surgery, Medical Faculty, Universitas Sam Ratulangi - Prof. Dr. R. D. Kandou Hospital, Manado, Indonesia; b General Practitioner at Pancaran Kasih Hospital, Manado, Indonesia

**Keywords:** Neglected posterior hip dislocation, Total hip replacement, Skeletal traction, Harris hip score

## Abstract

**Introduction and importance:**

Neglected posterior hip dislocations in adults are rare, particularly when untreated for years. In developing nations, patients often rely on traditional bone setters, leading to delayed diagnosis and increased complications. Adult hip dislocations carry a higher risk of avascular necrosis and require complex treatments. This case illustrates a five-year delayed posterior hip dislocation with a false acetabulum, managed with skeletal traction and total hip replacement. This case report is written in compliance with the SCARE guideline.

**Case presentation:**

A 64-year-old Minahasa woman presented with six years of hip pain, gait issues, and functional limitations post-fall. Initially treated by a bone setter, the patient had an 8 cm limb length discrepancy, restricted hip motion, and a Harris Hip Score of 39 %. Imaging showed a high-displaced hip with a false acetabulum and acetabular fracture. Treatment involved a two-staged procedure: skeletal traction and reconstruction followed by cemented total hip replacement, resulting in significant recovery and a Harris Hip Score of 90 % after four years.

**Clinical discussion:**

Chronic hip dislocations present challenges due to fibrous tissue and muscle contractures. Skeletal traction facilitated the reduction of the dislocation and provided stabilization prior to the replacement surgery. Total hip replacement was required due to the extent of joint damage. A Staged management skeletal traction and reconstruction before surgery improve outcomes in such cases, making total hip replacement an optimal solution for chronic dislocations.

**Conclusion:**

Total hip replacement is effective for neglected hip dislocations, restoring function and mobility, as demonstrated by this case's successful long-term outcome.

## Highlights

• Neglected posterior hip dislocations raise risks for avascular necrosis.
• Initial traditional treatment failed, leading to complex surgical interventions.
• Two-stage THR improved the Harris Hip Score from 39 % to 90 % post-surgery.
• Cemented total hip replacement restored function after six years of dislocation.
• THR is effective for neglected hip dislocations, achieving significant recovery.

## 1 Introduction

Posterior hip dislocations are rare in adults but more common in developing nations due to traditional bone setters' belief in them, highlighting the importance of timely treatment [1]. The likelihood of avascular necrosis of the femoral head increases with prolonged dislocation, as the retinacular artery of the medial circumflex femoral artery may become kinked. Immediate reduction or surgery is therefore crucial [2]. Sciatic nerve injury is the most common nerve complication associated with posterior hip dislocations (10% of cases), followed by peroneal branch and lumbosacral roots [3]. Treatment approaches for posterior hip dislocation differ between adults and children. In pediatric patients, dislocations require less force and have lower osteonecrosis rates due to more malleable tissues, cartilage, and improved vascularity, enabling quicker recovery [1].

Few studies have compared the outcomes of treatment for adult neglected posterior hip dislocations, given their rarity. Neglected hip dislocations become increasingly challenging to manage over time as the acetabulum fills with fibrous tissue, rendering closed reduction impossible. There remains ongoing debate regarding the optimal treatment approach for neglected hip dislocations. Various surgical techniques have been documented, including hemiarthroplasty, total hip replacement, hip arthrodesis, the Girdlestone procedure, and sub-trochanteric osteotomy [4]. Total hip arthroplasty is often recommended for chronic posterior dislocation due to risks such as iatrogenic femoral shaft fracture, unstable reduction from fibrous tissue overgrowth in the acetabulum, and avascular necrosis of the femur head. However, a trial of reduction of neglected or unreduced hip dislocation can be attempted [2].

We report a case of chronic dislocation of the left hip that was initially treated by the traditional bone setters but presented in our facility due to pain in the hip, difficulty with walking, and limitation of the activity of daily living. The aim of this study was to describe the evaluation and treatment method used in a complicated case of neglected hip dislocation, encompassing the entire therapeutic arsenal available to the orthopedic surgeon, and to review the existing literature [5]. This case report is written in compliance with SCARE guideline [6].

## 2 Case presentation

A 64-year-old female housewife from Minahasa with a six-year history of falling from a height presented to our facility in July 2018 with complaints of persistent left hip pain, limping, difficulty walking, and limitations in activities of daily living. Following the initial injury, she was treated by a traditional bone setter. The patient reported no significant medical or surgical history and no current or past medications.

**Fig. 1:**
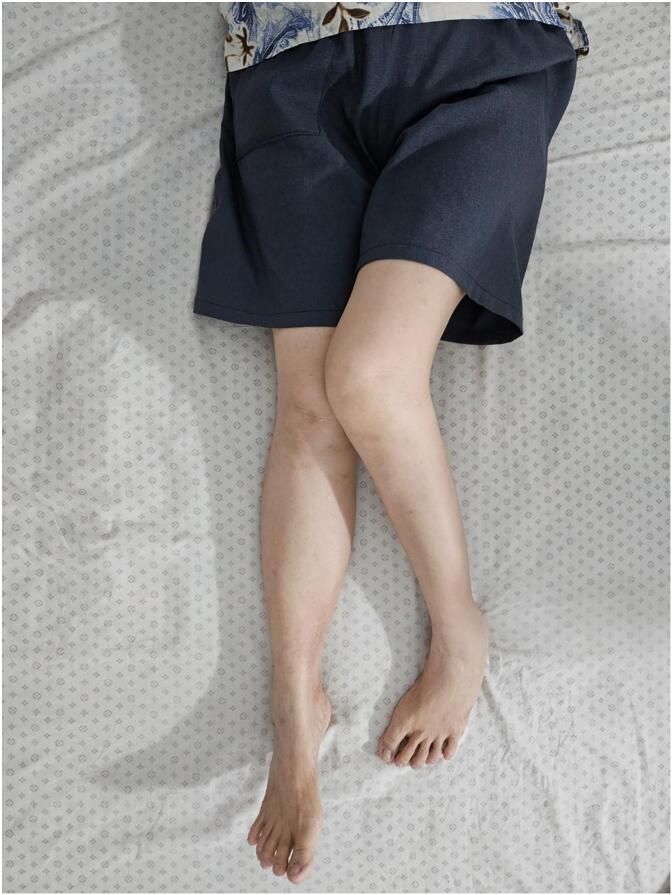
Chronic hip dislocation in the supine resting position.

**Fig. 2:**
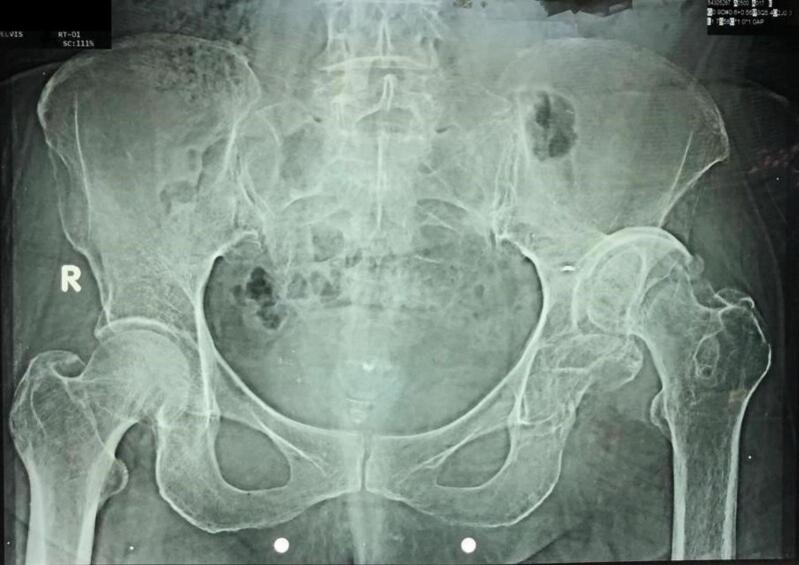
A plain radiograph shows a neglected left posterior hip dislocation with a false acetabulum and a fracture of the acetabular floor.

Clinical examination revealed a short-limb antalgic gait and a decreased range of motion in the left hip. The affected limb was shortened, with a limb length discrepancy (LLD) of 8 cm, fixed in adduction, flexion and internal rotation. The Harris Hip Score (HHS) was 39 %. (Fig. 1) Radiographic evaluation confirmed a high dislocation of the left hip with a false acetabulum in the supra-acetabular region and an acetabular floor fracture classified as Type 4 by Thompson and Epstein classification system (Fig. 2).

The patient underwent a two-stage surgical procedure.

**Fig. 3:**
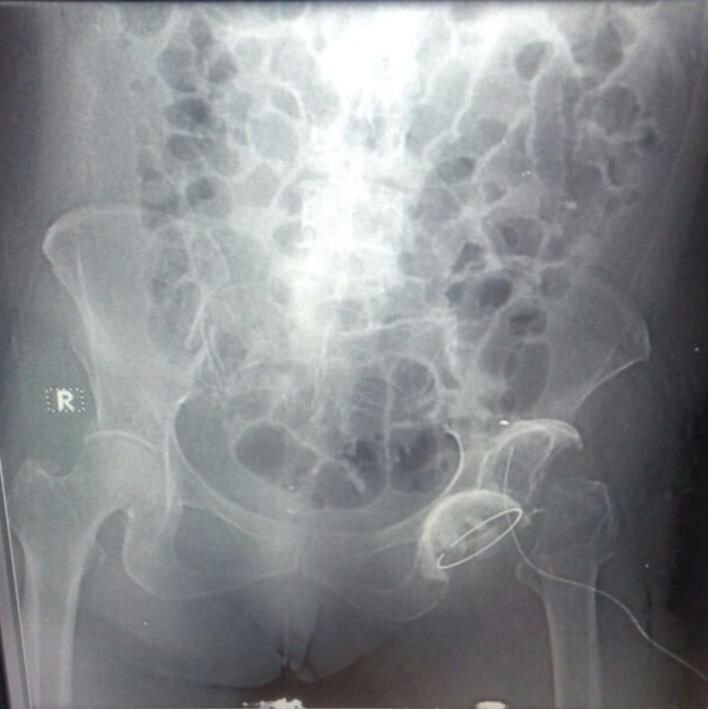
Plain radiograph after the first stage of surgery shows the application of a left acetabular cap and osteotomy of the left femoral neck.

• First Stage: The hip was approached posteriorly. The findings during the first stage of surgery revealed a false acetabulum in the supra-acetabular region, and the femoral head was noted to be globally soft. Extensive soft tissue release was performed around the femoral head and acetabular floor, followed by osteotomy of the femoral neck as low as feasible. The acetabular floor was reconstructed, and an acetabular cap was applied. Skeletal traction was initiated at 8 kg and gradually increased to 12 kg, maintained for 10 days. Postoperative radiographs from the first stage are shown in Fig. 2.
• Second Stage: The patient underwent cemented total hip arthroplasty. Postoperative radiographs from this stage are shown in Fig. 3.

Neurological and vascular examinations were intact both preoperatively and postoperatively. The patient had an uneventful recovery and was discharged two weeks postoperatively on partial weight-bearing with bilateral axillary crutches. The LLD improved to +1 cm.

**Fig. 4:**
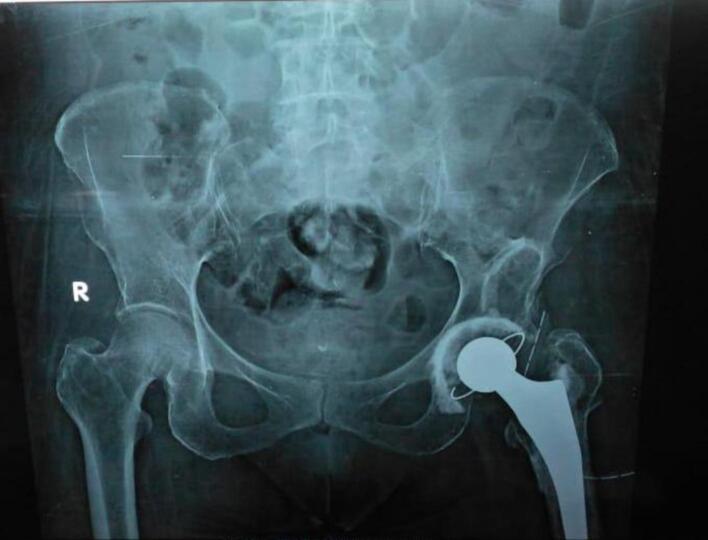
The plain radiograph shows left total hip replacement after surgery.

Follow-up evaluations were conducted at 1 week, 6 week, 3 months, and 6 months postoperatively. At the most recent follow-up in April 2022, the patient had a Harris Hip Score of 90 %. A pelvic anteroposterior (AP) radiograph was performed during the last follow-up is shown in Fig. 4.

## 3 Discussion

One of the orthopedic emergencies is posterior dislocation of the hip, which requires immediate attention due to the critical role of timing in its progression. Prolonged dislocation increases the risk of avascular necrosis of the femoral head. The risk rises from less than 10 % if reduction occurs within 6 h of the injury to over 50 % if delayed beyond this timeframe. Additionally, secondary osteoarthritis is a potential long-term complication following posterior hip dislocation. However, as illustrated in this case, patients in developing countries often delay seeking treatment at the hospital. Even when they do, local bone setters may inadequately manage the dislocation previously [1,2].

Hip dislocations are rare and typically result from high-energy trauma due to the stability of the surrounding soft tissues and the joint's natural congruency [5]. The bony structure and strong ligaments of the hip joint enable it to withstand significant mechanical stress. Key anatomical features that enhance the hip's stability include the labrum, joint capsule, muscles, surrounding ligaments, and the depth of the acetabulum [7]. The anterior iliofemoral ligament and the posterior ischiofemoral ligament are the primary ligaments responsible for stabilizing the joint against directional forces. Due to the strength of the anterior ligaments, posterior dislocation accounts for approximately 90 % of hip trauma cases [8].

Treating neglected hip dislocation becomes increasingly challenging over time as delayed presentation can lead to complications such as limb shortening, gait abnormalities, and difficulties with daily activities. The acetabulum may become filled with fibrous tissue, and soft tissue contracture can develop, resulting in the limb being fixed in flexion, adduction, and internal rotation [2]. In this case, the patient presented six years after the initial injury, having previously received treatment from a local bone setter. The neglected posterior dislocation resulted in a shortened limb (limb length discrepancy of 8 cm) fixed in adduction, flexion, and internal rotation, with a preoperative Harris Hip Score (HHS) of 39 %.

The treatment of neglected hip dislocations remains a topic of ongoing debate, with various surgical techniques proposed. Options include hemiarthroplasty, total hip replacement, hip arthrodesis, the Girdlestone procedure, and subtrochanteric osteotomy [4]. Beebe emphasized the importance of promptly reducing the femoral head back into the acetabulum. In the emergency department (ED), every hip dislocation is subjected to an attempt at closed reduction under deep conscious sedation with propofol, except in cases with relative contraindications, such as fractures of the femoral neck or shaft, prior to advanced imaging [8].

A study by Olasinde indicates that the initial treatment for neglected posterior dislocation of the hip involves heavy trans-tibial skeletal traction, which is progressively increased over a two-week period to stretch the contracted soft tissues [2]. Similarly, Kumar et al. and Jain described a case involving a patient who presented 1.5 years after the initial injury, having previously received treatment at a local bone setter facility. Due to the delayed presentation, the patient first underwent heavy trans-tibial skeletal traction, which was gradually increased over two weeks to stretch the contracted soft tissues. In a study involving eighteen patients, Jain and Kumar et al. found that open reduction was necessary after the failure of strong trans-tibial skeletal traction [1,4]. Selimi et al. recommended the use of trans-tibial skeletal traction as a preoperative procedure for patients with hip dislocations that were more than a year old. This approach is intended to stretch the tense and contracted hip muscles, facilitating a more effective reduction [9].

For longstanding posterior dislocations with acetabular fractures, studies by Garret et al. suggest total hip replacement (THR) as the most effective treatment. Garret recommended THR for dislocations misaligned for over three months, particularly those classified as Type IV (acetabular rim and floor fractures) or type V (femoral head fracture, with or without additional fractures) [10]. Jain found primary THA to yield optimal outcomes for neglected dislocations associated with Type II or higher acetabular fractures [1]. In our case, the patient presented six years post-injury with radiological findings of posterior hip dislocation, a false acetabulum, and an acetabular floor fracture. The patient underwent a two-stage procedure. The first stage involved extensive release of soft tissues around the femoral head and acetabular floor was performed. The femoral head was found to be globally soft, and the neck was osteomized as low as feasible. The acetabular floor was reconstructed, and an acetabular cap was applied. Skeletal traction was initiated at 8 kg and progressively increased to 12 kg over 10 days to stretch contracted soft tissues. The second stage procedure was cemented total hip replacement.

In their study, Ilyas and Rabbani performed total hip arthroplasty (THA) to treat grades III and IV dislocations and observed a significant improvement in the patients' conditions [11]. In their case series, Jain et al. reported an increase in the preoperative Harris hip scores of three patients, rising from 27 to 42, and ultimately to between 81 and 91 [1]. According to a published report by Olasinde, the patient underwent bipolar hemiarthroplasty due to financial constraints and a lack of total hip replacement (THR) instruments at the hospital. Postoperatively, the Harris Hip scores improved from 40.6 % to 90.8 % [2]. In our case, the preoperative Harris Hip scores rose from 39 % to 90 % in 6 months post-surgery. We, therefore, recommend that a total hip replacement is a reasonable alternative for the patient presenting with a neglected posterior dislocation of the hip more than a year.

## 4 Conclusion

Total hip replacement (THR) emerges as the preferred treatment for neglected posterior hip dislocation lasting more than a year which resulted in significant functional improvement and a high Harris Hip Score postoperatively.

## Author contribution

First author

‐ Conceptualization
‐ Investigation
‐ Resource
‐ Methodology
‐ Supervision
‐ Funding Acquisition
‐ Methodology
‐ Writing-review & editing

Second author

‐ Visualization
‐ Data curation
‐ Writing-review & editing
‐ Project Administration
‐ Supervision
‐ Funding Acquisition

Third author

‐ Investigation
‐ Formal analysis
‐ Writing – review & editing
‐ Validation
‐ Funding Acquisition

Fourth author

‐ Resources
‐ Visualization
‐ Validation
‐ Funding Acquisition

Fifth author

‐ Methodology
‐ Writing – original draft
‐ Validation
‐ Funding Acquisition

Sixth author

‐ Writing – original draft
‐ Software
‐ Project administration

## Ethical approval

An ethical exemption for this study was obtained from the institutional Research Ethics Committee. All procedures involving human participants were conducted in accordance with the ethical standards of the institutional and/or national research committee, as well as the 1964 Helsinki Declaration and its later amendments or comparable ethical standards.

## Guarantor

Andriessanto Ceelvin Lengkong.

## Patient perspective

The patient expressed significant concern about her ability to return to normal daily activities following her injury. She reported that her left hip pain had not only affected her mobility but also limited her ability to care for her family and engage in social activities, which she valued greatly. The patient described feeling frustrated and anxious about her condition, particularly after initially relying on a traditional bone setter, as she felt her pain was not adequately addressed.

During consultations, the patient voiced her appreciation for the medical team's approach, highlighting the importance of being listened to and involved in her treatment decisions. She stated, she wants to walk again like she used to and take care of her household without pain. This desire for independence motivated her to adhere to the recommended rehabilitation plan and follow-up appointments.

Throughout her treatment, the patient indicated a strong preference for clear communication about her progress and any potential side effects of treatments. She reported feeling empowered when she was informed about her condition and the steps needed to improve her quality of life.

## Research registration number

This study does not meet the criteria for 'First in Man' and therefore does not require registration.

## Consent for publication

Written informed consent has been obtained from the patient for publication of this case report and accompanying images. A copy of the written consent is available for review by the Editor-in-Chief of this journal upon request.

## Funding

No external funding was received.

## Conflict of interest statement

The authors declare that they have no known competing financial interests or personal relationships that could have appeared to influence the work reported in this paper.
